# A seed or soil problem in early endometriosis: stromal cell origin drives cellular invasion and coupling over mesothelial cell origin

**DOI:** 10.1016/j.xfss.2024.08.001

**Published:** 2024-08-08

**Authors:** Virginia-Arlene Go, Jeffery Chavez, Randal D. Robinson, Bruce J. Nicholson

**Affiliations:** a Department of Obstetrics and Gynecology, Division of Reproductive Endocrinology and Infertility, University of Texas Health San Antonio, San Antonio, Texas; b Department of Biochemistry and Structural Biology, University of Texas Health San Antonio, San Antonio, Texas

**Keywords:** Endometriosis, primary mesothelial cells, invasion, gap junction coupling

## Abstract

**Objective:**

To study the role of the mesothelial cells in early endometriosis lesion formation by assessing in vitro cell-to-cell communication and invasion of endometrial cells across a mesothelial cell monolayer, with both cell types derived from both patients with endometriosis and control patients.

**Design:**

Laboratory-based experimental study.

**Setting:**

University hospital and laboratory.

**Patient(s):**

Consenting reproductive-age women who underwent laparoscopy for gynecologic reasons and were confirmed to have either endometriosis with pathology tissue diagnosis (n = 8) or no endometriosis n = 8) at the time of surgery.

**Intervention(s):**

Primary stromal cells cultured from endometrial pipelle biopsies and primary mesothelial cells cultured from peritoneal explants were used in transmesothelial invasion assays and gap junction coupling assays.

**Main Outcome Measure(s):**

Comparison of potential for lesion formation, using in vitro models, of both primary endometrial and mesothelial cells from patients with endometriosis and control patients, establishing the former as the primary disease driver.

**Result(s):**

When comparing mesothelial cells from control patients with those from patients with endometriosis, there was no significant difference in the amount of stromal cell invasion across either barrier. In contrast, when comparing stromal cell origin, the amount of invasion by endometriosis stromal cells was greater than control stromal cells regardless of whether the mesothelial cell monolayer was derived from patients with the disease or control patients. Additionally, primary mesothelial cells induced more gap junction coupling, a requirement for invasion, in stromal cells from patients with endometriosis than control patients, again independent of mesothelial origin. The notable exception was mesothelial cells derived from endometriotic lesion-affected areas that showed depressed ability to support invasion.

**Conclusion(s):**

Although both endometrial and mesothelial cells need to function for establishment of endometriosis lesions, the endometrium seems to be the key player, serving as an ideal target for diagnostic strategies and therapeutic intervention. While this notion is consistent with previous studies, to our knowledge, we are the first to directly test both primary mesothelial and endometrial cells from patients with endometriosis and control patients to compare propensities for mesothelial invasion.

Endometriosis is a pervasive gynecologic disease affecting approximately 1 in 10 reproductive-age women overall (1, 2). Among asymptomatic women seeking elective sterilization, endometriosis is found in approximately 1%–7% of patients (1). The prevalence increases among those with reported symptoms such as pelvic pain and infertility (2). In the United States, endometriosis is diagnosed in 35%–50% of women with pelvic pain and up to 50% of women with unexplained infertility (3, 4). Patients with endometriosis have been found to have lower fecundity rates and decreased success with assisted reproductive technology (4–6).

The pain associated with endometriosis may present during menstrual cycles, intercourse, or bowel movements or simply without provocation. Additionally, endometriosis may present with abdominal bloating and/or irregular bleeding; symptoms often last throughout a woman’s reproductive life.

Beyond the physical and emotional impact to those with the disease, endometriosis poses a financial burden to our society. This presents in the form of emergency room visits, excessive laboratory tests and imaging studies during the average 6.7-year delay to diagnosis, lost wages from missed work, and ultimately a costly surgery as the gold standard of diagnosis (7). Endometriosis costs the United States alone $80 billion per year between prolonged healthcare costs and loss of productivity (8).

Despite the high prevalence of disease and burden to society, the pathophysiology of early disease development remains poorly understood. Endometriosis is a complex systemic disease associated with extrauterine endometrial-like glands and stroma; however, the question remains of how the ectopic endometrial sites develop. The popular Sampson’s theory (9) suggests that endometrial cells travel through the fallopian tubes into the peritoneal cavity by retrograde menstruation. However, the number of individuals who experience retrograde menstruation far outweighs the number of individuals who have endometriosis (10), indicating that there are additional factors predisposing approximately 10% of individuals to the disease. There are also rare reports of visualized endometriosis lesions in premenarchal patients and male patients that are likely best explained by differentiation of stem cells or developmental remnants (11–13). In addition to the previously discussed retrograde menstruation, stem cell theory, and müllerian remnants, other popular endometriosis pathophysiology theories include coelomic metaplasia, vascular/lymphatic dissemination, and direct transplantation of endometrial tissue. The theory of coelomic metaplasia suggests that there may be spontaneous metaplastic change in mesothelial cells derived from the coelomic epithelium (located in the peritoneum and the pleura), possibly induced by exposure to menstrual effluent or other stimuli such as estrogen (14–16). This may help explain cases of endometriosis in the thoracic cavity that retrograde menstruation should not reach (15, 16). The theories of vascular or lymphatic dissemination stem from the notion of an invasive endometrial cell, which may extend to intravasation and extravasation similar to metastatic tumor spread to lymph nodes and distant sites (17–20). Lastly, direct transplantation or surgical dissemination may be supported by cases of endometriosis lesions noted in cesarean section scars and after other uterine surgery where the endometrial tissue may be iatrogenically seeded into other tissues through techniques such as morcellation (21–23).

No single theory completely explains all cases of endometriosis; thus, a gap remains in understanding the origin of endometriosis and the driving factor(s) in disease development. Do patients with endometriosis have an endometrium that is particularly invasive or peritoneum that is particularly susceptible to lesion formation, or is there some sort of combination and balance that is disrupted with disease? In other words, we still do not understand if disease propensity lies within the seed (endometrium) or soil (peritoneum), or perhaps both, in coordination.

Through previous work, we have demonstrated in vitro that human endometrial cells invade more across an established mesothelial cell (LP9 cell line) monolayer when taken from patients with endometriosis than from controls. Similarly, LP9 mesothelial cells induced more coupling in stromal cells from patients with endometriosis than those from control patients, a step that we also showed to be required for invasion to occur (24). Existing literature supports a principal role of endometrial cells in endometriosis lesion development. A previous study by Lucidi et al. (25) concluded that endometrial stromal cell (ESC) binding to mesothelial cells was dependent primarily on the source of ESCs; however, this question has not been addressed by studying ESC communication with the mesothelium and invasion itself. Furthermore, this has not been tested with primary mesothelial cells (PMCs) taken from both control patients and those with endometriosis (25).

The goal of this project was to directly compare the influences of both mesothelial and endometrial cell origins in the invasive steps needed to initiate endometriosis lesion formation using an in vitro model of invading endometrial cells across a mesothelial cell monolayer, with the ability to vary the source of each cell type from either patients with endometriosis or control patients.

## MATERIALS AND METHODS

### Cell culture

Primary mesothelial cells were isolated from human peritoneal biopsies obtained from consenting patients undergoing laparoscopic surgeries for gynecologic indications. All procedures were conducted in accordance with institutional ethical guidelines, and informed consent was obtained from participants under institutional review board protocol number 20070728HR from UT Health Science Center San Antonio. At the time of laparoscopic surgery, patients were noted to have endometriosis or no disease, a conclusion confirmed on final pathology report.

**FIGURE 1:**
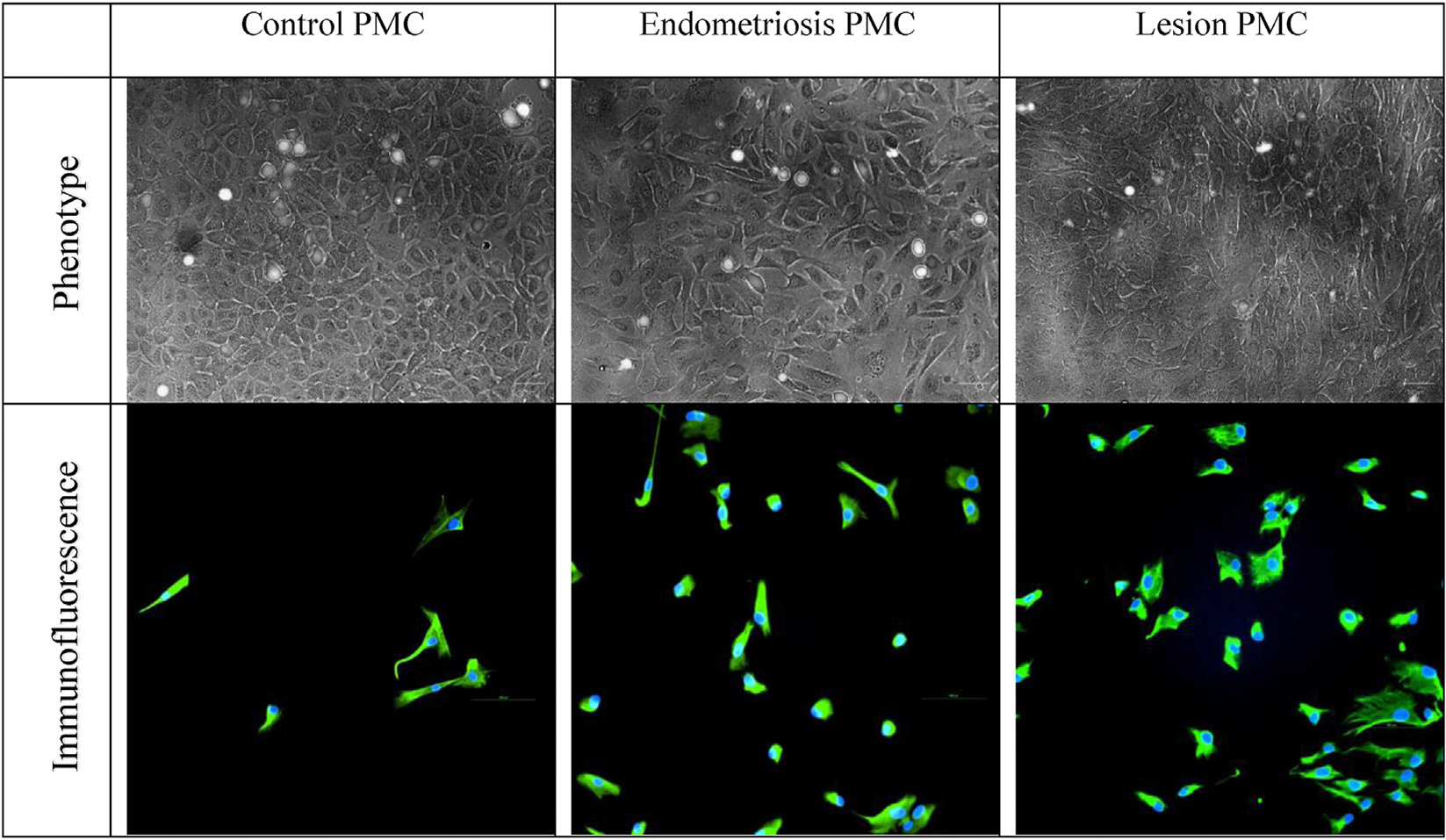
Mesothelial cell characterization. Primary peritoneal mesothelial cells were identified by their phenotypic cobblestone appearance when in a monolayer (*top* row; Bar = 50 *μ*m) and Calretinin immunofluorescence labeling in lower-density cultures (*bottom* row; Bar = 100 *μ*m). Mesothelial cells from control patients, patients with endometriosis, and lesion areas had similar cell characterization overall. PMC = primary mesothelial cell.

The PMCs were cultured from peritoneal tissue explants similarly to the protocol published by Stylianou et al. (26). Briefly, peritoneal biopsies were sectioned into approximately 1-mm^2^ segments. Peritoneal tissue explants were placed onto 6-cm Matrigel (Cat#354230 - CorningInc., Corning, NY) coated plates and partially submerged in LP9 media. LP9 media consisted of MCDB 131 (Gibco #10372–019)/Medium 199 (Gibco #12340–030 - Thermo Fisher, Waltham, MA), 15% fetal bovine serum (FBS) (USDA Approved Heat Inactivated Cat#FB-02 - Omega Scientific, Tarzana, CA), 1% antibiotic/antimycotic (Gibco Cat#15240–062), 1-mM sodium pyruvate (Gibco #11360–070), 1% GlutaMAX (100X, Gibco #35050–061), 20 ng/mL of human epidermal growth factor (Sigma #E9644 - Sigma Aldrich, St. Louis, MO), and 0.4 ng/mL of hydrocortisone (Sigma #H0888). These plates were then incubated at 37°C and checked daily to assess cellular growth. Once the cells were confluent, Accutase (Corning Cat#25–058-CI) was used to remove the cells for utilization in various cell characterization and assay experiments. In addition to their phenotypic cobblestone appearance, consistent with descriptions by Stylianou et al. (26), Calretinin (Cat#ab92341 - Abcam, Cambridge, MA) immunofluorescence labeling was used to confirm the mesothelial cell identity of the isolated cells (Fig. 1). These PMCs were then used in both transmesothelial invasion and gap junction coupling assays.

Similarly, primary ESCs were isolated from pipelle endometrial biopsies obtained at the time of laparoscopy, again from consenting patients with and without endometriosis under institutional review board protocol #20070728HR. The endometrial tissue was dissociated by shaking in 5 mg/mL of collagenase and 2.5 mg/mL of DNase in Hanks Balanced Salt Solution at 37°C for 1 hour. On the basis of the methods developed by Kirk and Irwin (27) and used in previous studies, primary ESCs were isolated using a combination of straining (45-*μ*M nylon filter), differential sedimentation (epithelial cell clusters sediment faster), and differential attachment (epithelial cells adhere less well to culture plates) (28, 29). Stromal and epithelial cell identity was confirmed with immunofluorescence staining with vimentin (MA1–10459 from Thermo Fisher Scientific [Waltham, MA] and NBP1–92687 from Novus Biologicals [Centennial, CO]) and cytokeratin 7 (#ab181598- Abcam, Cambridge, UK). Stromal cells were grown in Dulbecco’s Modified Eagle Medium/Nutrient Mixture F-12 (Gibco #11320–033 - Thermo Fisher, Waltham, MA), 10% FBS (USDA Approved Heat Inactivated Cat#FB-02, Omega Scientific, Tarzana, CA), 1% antibiotic/antimycotic (Gibco Cat#15240–062 - Thermo Fisher, Waltham, MA), and 10 *μ*g/mL of insulin (Cat#I0516 - Sigma Aldrich, St. Louis, MO).

### Transmesothelial invasion assay

A 3-dimensional in vitro model of transmesothelial invasion has been previously described (29–31). Briefly, the isolated PMCs were grown to confluence over 48–72 hours in 24-well invasion chamber inserts containing growth factor–reduced Matrigel, coated on 8-*μ*m pore membranes (Corning, NY). Patient-derived ESCs were labeled with the lipophilic dye DiO (Invitrogen V22886) from Thermo Fisher, Walthan, MA, trypsinized and counted, before dropping onto the confluent layer of PMCs in the prepared inserts (approximately 20,000 cells per insert). Stromal medium was placed below the insert. In some experiments, invasion was measured under a 1% serum gradient (serum-free stromal medium on top and stromal medium with 1% FBS on the bottom).

After 24 hours of incubation, noninvading cells on the upper surface of the insert were mechanically removed. Invading cells on the bottom of the membrane insert were stained with 4′,6-diamidino-2-phenylindole, and 9 fields were counted using an Inverted Nikon 2000 fluorescence microscope with an objective of ×20, confirming in each case that the 4′,6-diamidino-2-phenylindole–stained nuclei were associated with DiO staining. Invasion assays for each cell type were performed in triplicate, and an established stromal cell line (tHESCs) was used in each experiment to provide standardization so that results could be compared across experiments.

### Gap junction coupling assay

To assess gap junctional intercellular coupling (GJIC) between cells, a dye transfer assay, as previously described by Chen et al. (24), was performed using the gap junction–permeable dye, calcein AM (Invitrogen C3100MP - Thermo Fisher, Waltham, MA). Acceptor cells were grown to confluence in a 96-well plate. The medium was changed to assay medium (phenol red–free Dulbecco’s Modified Eagle Medium, sodium pyruvate, and 5% FBS) immediately before the assay. Donor cells were grown in separate wells and then incubated for 20 minutes with 10-*μ*M calcein AM (Invitrogen), a membrane permeable dye that becomes fluorescent and membrane impermeable on cleavage by intracellular esterases but now rendered permeable to gap junctions. After rinsing, trypsinization, and adding assay medium, approximately 2,500 calcein-labeled donor cells per well were dropped onto the acceptor cell monolayer, and the level of calcein dye transfer between donor and acceptor cells was captured by fluorescent microscopy. For homocellular GJIC, ESCs or PMCs were dropped onto acceptor cells of the same type. For hetero-cellular GJIC assays, ESCs were dropped onto PMC acceptor cells. Fluorescent, bright-field, and phase contrast images were captured on an automated Operetta microscope (PerkinElmer, Waltham, MA) every 30 minutes for approximately 2.5 hours. A computer program developed with PerkinElmer allowed identification of all cells on the plate (from phase contrast images), original donors (defined on the basis of the level of fluorescence, 5–15 per field), and dye-filled recipients (at lower fluorescent intensity than donors). Data were expressed as the increase per hour in the number of fluorescent acceptor cells/donor cell (acceptor/donor ratio), on the basis of a linear regression fit of the data (typically with R^2^ of 0.8–0.99). Unpaired, 2-tailed *t*-tests were performed on GraphPad Prism for statistical analysis across all experiments.

## RESULTS

A total of 16 reproductive-age patients were included in the study, 8 with endometriosis and 8 control patients (Table 1). Primary mesothelial cells were cultured from 14 of the patients, 7 with and 7 without endometriosis. In addition to standard peritoneal sampling from the 14 patients, separate tissue biopsies from lesion-affected areas were obtained for 3 of the 7 patients with endometriosis and cultured separately.

Endometrial stromal cells were derived from the frozen biorepository established in the laboratory and included 4 control patients and 4 patients with endometriosis from the total patient population, confirmed as described earlier. In most cases, ESCs were collected from the same patients who provided the PMCs (Table 1). In some assays, cells were used before freezing; however, no differences were observed between fresh and frozen-thawed cells.

### Transmesothelial invasion

In vitro invasion assays provide a measure of the inherent invasive potential of endometrial cells on interaction with a mesothelial monolayer and of the susceptibility of the mesothelial monolayer to invasion. The assays mimic the complex microenvironment encountered by shed endometrial cells within the peritoneal cavity, thereby facilitating the assessment of invasion-associated signaling pathways (32). This allows for a direct comparison of the invasive capacity of the samples of both endometrial and mesothelial origin from control patients and those with endometriosis. Although we do not include any immune components, the object of this assay was to examine the earliest stages of lesion formation, before significant induction of the inflammatory processes that arise later during disease progression.

**FIGURE 2:**
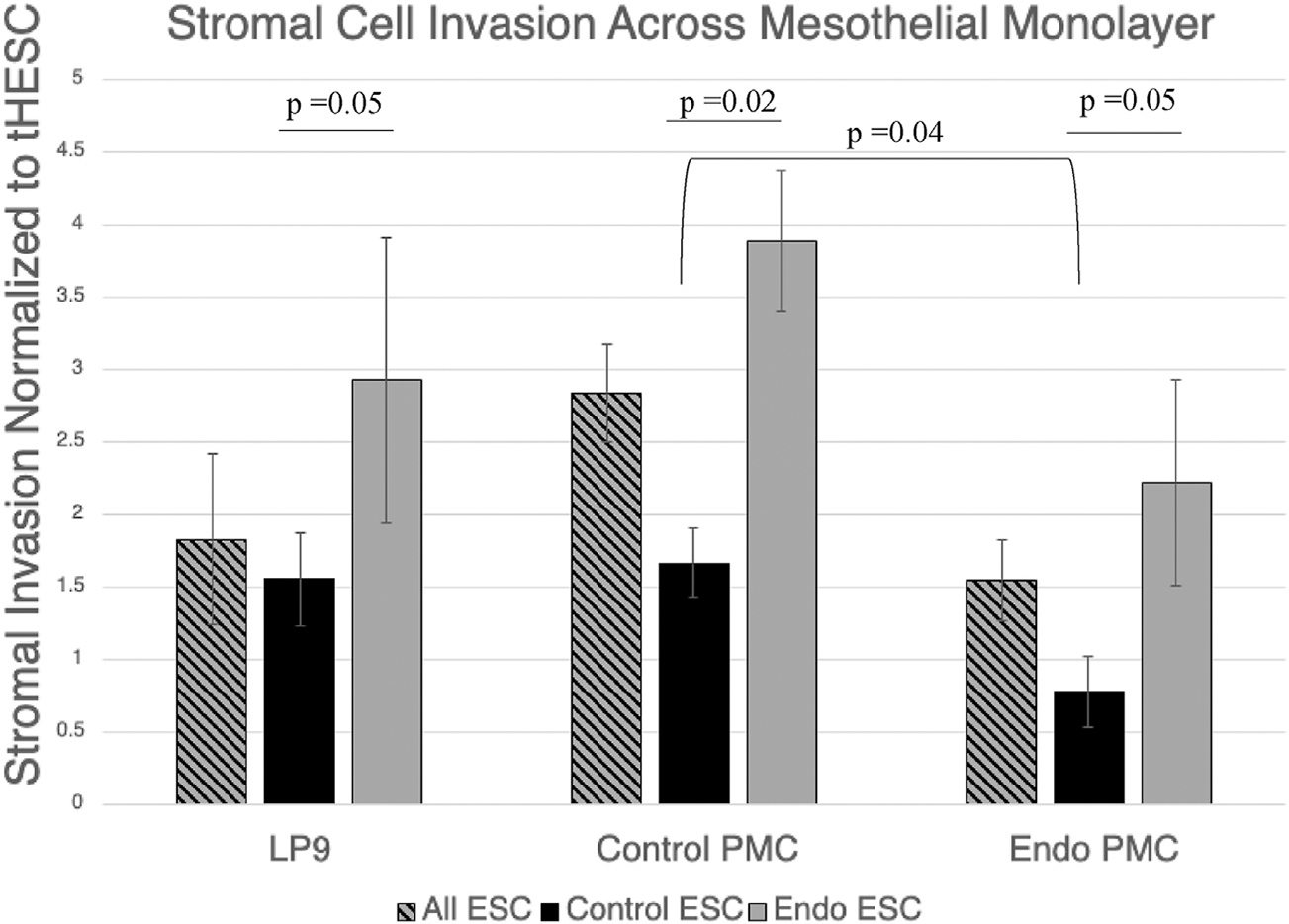
Control and endometriosis stromal cell invasion across mesothelial cell monolayers of various origins. Independent of mesothelial origin, endometrial stromal cells (ESCs) from patients with endometriosis showed an approximately twofold greater invasion than those from controls. The primary mesothelial cells (PMCs) of patients with endometriosis tended to allow less invasion, particularly of control ESCs. LP9 (established mesothelial cell line), N = 13; Control PMC, N = 4; and endometriosis PMC (Endo PMC), N = 3. Control ESC, N = 2; endometriosis ESC (Endo ESC), N = 2. Invasion studies conducted under 1% serum gradient. Standard *error bars* and unpaired, 2-tailed *t*-test *P* values shown.

As previously demonstrated by Chen et al. (24), we also observed greater invasion of ESCs from patients with endometriosis across an established mesothelial cell monolayer of LP9 cells than that from control patients (Fig. 2). In contrast, we noted no impact of PMC origin on the level of overall stromal invasion (Fig. 2). In comparisons of PMCs from different origins, the most significant difference was noted when stratifying stromal cells by their origin from either control patients or those with endometriosis. Endometrial stromal cells from patients with endometriosis showed a twofold (*P*=.05) greater invasion than those from control patients across the established PMC cell line (LP9 cells—Coriell mesothelial cell strain LP-9 Cat#AG07086). When we used similar conditions to test PMCs from control patients and those with endometriosis, we saw similar 2.5- and 3-fold (*P*=.02 and *P*=.05, respectively) increases in endometriosis over control ESCs, respectively (Fig. 2). Stromal cells from patients with endometriosis invaded more across a mesothelial cell monolayer, regardless of the mesothelial cell origin. Thus, the enhanced invasion of ESCs from patients with endometriosis was independent of the origin of the PMCs.

Our compiled data comparing primary cells from both the endometrium and mesothelium of control patients and those with endometriosis make a compelling case that stromal cell origin is the primary determinant of invasion in early endometriosis development. Mesothelial cell origin appears to have minimal influence. In fact, mesothelial cells from patients with endometriosis tended to allow less invasion than those from control cells in comparisons of control, endometriosis, or combined ESCs, although this trend only reached significance for control ESC comparisons (*P*=.04) (Fig. 2). This may suggest that patients who have sufficient disease burden to diagnose endometriosis already have a peritoneal cavity that has undergone some sort of transformation or inflammatory response that may compromise mesothelial cell function.

**FIGURE 3:**
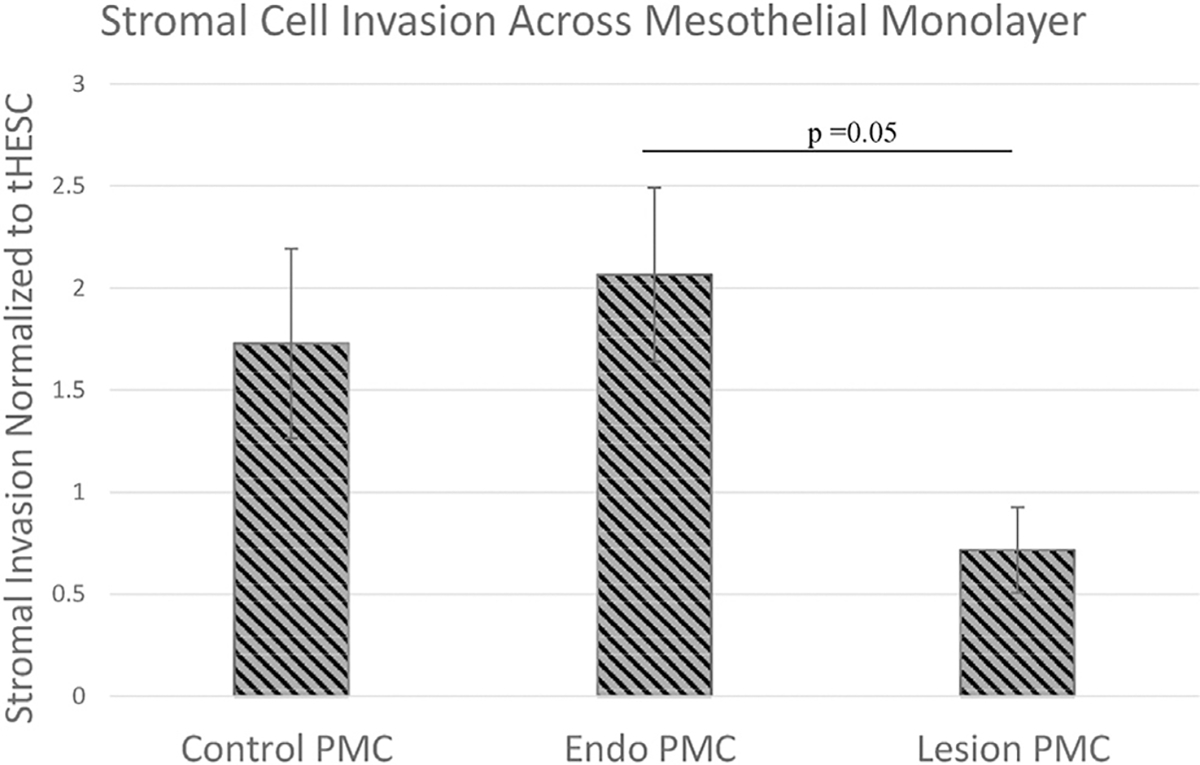
Overall stromal cell invasion across mesothelial cell monolayers of varied origins. Primary mesothelial cells (PMCs) derived from lesion areas of patients with endometriosis allowed less invasion compared with those from the healthy peritoneal areas of either patients with endometriosis or control patients. Control PMC, N = 7; endometriosis PMC (Endo PMC), N = 7; and Lesion PMC, N = 3. Stromal cells, N = 7. Invasion studies conducted under no serum gradient. Standard *error bars* and unpaired, 2-tailed *t*-test *P* values shown. ESC = endometrial stromal cell.

Further supporting this idea of debilitated mesothelial cell function in endometriosis, we found that mesothelial cells isolated from actual endometriotic lesion areas allowed less stromal cell invasion compared with mesothelial cells from the uninvolved peritoneal areas of the same patients with endometriosis (*P*=.05; Fig. 3). This would be consistent with a premise that although ESCs are the principal drivers of cellular invasion, the mesothelial cells do play an active role in promoting invasion that may be compromised in established endometriosis lesions. Supporting this conclusion, we have demonstrated previously that LP9 mesothelial cells actively induce gap junction coupling with ESCs, a requirement for invasion, and this effect is much higher in endometriosis-derived ESCs (24). Hence, we also aimed to investigate whether primary patient mesothelial cells also mediated this induction of gap junctions and whether this differed between control and endometriosis-derived PMCs.

### Gap junction coupling

**Figure 4:**
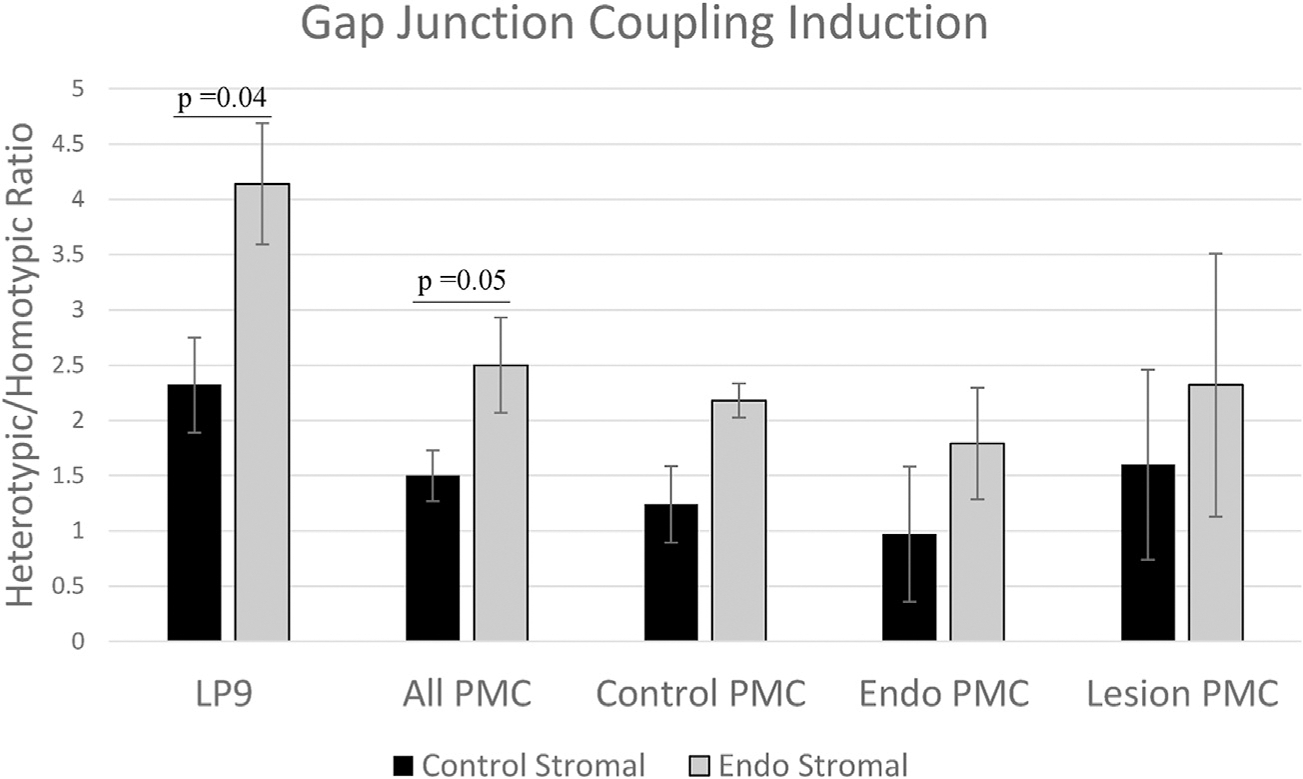
Stromal cell gap junction coupling induction by mesothelial cells of varied origins. All primary mesothelial cells (PMCs), either the LP9 cell line or primary cells from control or patients, showed greater induction of gap junctional intercellular coupling in endometrial stromal cells derived from patients with endometriosis than from control patients, although PMCs from lesion areas showed the least level of induction. LP9 (established mesothelial cell line), N = 4; All PMC, N = 14; Control PMC, N = 4; endometriosis PMC (Endo PMC), N = 4; and Lesion PMC, N = 2. Standard *error bars* and unpaired, 2-tailed *t*-test *P* values shown.

As reported previously, we also found that LP9 PMCs induced coupling in both control and endometriosis-derived ESCs (2.3- and 4.1-fold, respectively), although to a significantly greater degree in the latter (*P*=.04; Fig. 4). By comparison, primary PMCs from either control patients or those with endometriosis were found to only induce coupling in endometriosis ESCs (2.2- and 1.8-fold, respectively) and showed no induction of control ESCs. However, the relative increase in induction between endometriosis and control ESCs (1.8 ± 0.05-fold) was remarkably constant, independent of the PMC source (LP9 line or primary cells from control patients or those with endometriosis). The only exception, again, was PMCs derived from lesion areas, which showed a reduced difference between inductions of coupling between control and endometriosis ESCs of 1.45-fold (Fig. 4).

Thus, with respect to promotion of endometrial-mesothelial gap junction coupling, which we have shown to be required for invasion, this active role played by the mesothelium does not differ between control and patient mesothelial cells and, if anything, is partially compromised in the mesothelium of patients with disease. Rather, it is the responsiveness of the ESCs that shows consistent differences between control patients and those with endometriosis, strongly implicating the endometrium as the primary origin of disease. The reduced level of induction observed in lesion-derived PMCs is consistent with the hypothesis proposed earlier that mesothelial cells from lesion-affected areas may have compromised function and actually reduce the chance of additional endometrial invasion at the already affected areas of the peritoneal wall.

## DISCUSSION

Overall, mesothelial cells from patients with endometriosis and control patients did not differ in the level of permitted cellular invasion or coupling induction, 2 of the primary initial steps in endometrial lesion formation. In contrast, major and consistent differences were evident between endometrial cells from patients with endometriosis and control patients that explain the ability of the former to form lesions. This supports the idea that the endometrium is the driving factor in disease. An interesting difference was noted when mesothelial cells were taken from actual endometriotic lesion areas of the peritoneum and compared with the nonaffected areas of the peritoneum from the same patient. Lesion-derived tissue permitted less invasion, suggesting a compromise in mesothelial function that is required to support lesion formation once endometriosis invasion has already occurred. This may be correlated clinically because endometriosis lesions do not only cluster in one area but are also found throughout the peritoneal cavity, suggesting that the “seed” prefers, or even requires, “fresh soil” to properly implant and invade.

Although both seed and soil need to function for disease development, the endometrium seems to be the key player and the one that varies with disease state. This may also suggest a rationale as to why infertility is so often associated with endometriosis. It also indicates that the uterine lining itself will be the best target for diagnostic and ultimately therapeutic strategies. This notion is consistent with previous studies, including that of Lucidi et al. (25) who demonstrated that ESC binding to PMCs was dependent primarily on the source of ESCs (9). However, to our knowledge, the current study is the first to directly compare both PMCs and endometrial cells from patients with endometriosis and control patients, in terms of their ability to support endometriosis invasion, the first and critical step in lesion formation.

We acknowledge this is a simplistic in vitro model for endometriosis development that does not include immune components, which ultimately are an important aspect of disease development and maintenance. However, our goal was to assess these earliest stages of lesion formation and initial interaction between endometrium and peritoneum, before significant influence of various inflammatory markers, which arise later during disease. This in vitro model allows us to vary the sources of both stromal and mesothelial cells simultaneously to assess the impact of each and is a novel look into early disease formation. Because previous studies have demonstrated the significance of menstrual cycle stage in an endometriosis murine model, this proves to be an area for further investigation in our work (33). A potential limitation of our study is the lack of standardization and details of cycle stage for each patient. Often times, patients undergo gynecologic surgery while on oral contraceptive pills for ease of scheduling or intentionally while they are not menstruating. Either scenario could affect the molecular signatures of either stromal or mesothelial cell line and impact the potential for endometriotic lesions development. Although this does not negate our findings, it is an important point to note and consider in design for future studies.

The intricate interplay between primary mesothelial and endometrial cells holds significant implications for endometriosis pathogenesis. Coupling and invasion assays provide invaluable insights into the cellular interactions underpinning lesion formation. They provide a critical platform to identify the intricate signaling pathways that mediate the initial stages of lesion formation, which ultimately represents the best potential target for both diagnostic and therapeutic pursuits.

## CONCLUSIONS

A major question in the field of endometriosis that remains to be resolved is the degree to which lesion formation of endometrial cells in the peritoneum is primarily driven by more aggressive invaders (endometrial cells) or a more susceptible target (the mesothelium) or is it a combination of both. In the current study we definitively test this for the first time by using endometrial stromal cells (ESCs) and peritoneal mesothelial cells (PMCs) from endometriosis patients and control subjects in in vitro analyses of invasive potential. This also included assessing the induction of gap junction coupling between the two cells when they contact, which we have previously shown is more pronounced in endometriosis derived cells and is required for subsequent invasion.

While PMCs clearly play a role in facilitating invasion, as they cause a significant increase in gap junction coupling of ESCs both to themselves and PMCs, our results clearly show that the magnitude of this coupling induction, and the invasion of ESCs across a PMC barrier, is dictated by the source of the ESCs, and not the PMCs. PMCs from patients and controls induce similar levels of coupling, but this is consistently higher when the ESCs are derived from endometriosis patients. The same is true of invasiveness, where there is even a trend for PMCs from endometriosis patients to be LESS receptive to invasion. This is even more pronounced when the PMCs are derived from parts of the mesothelium that already has lesions, possibly suggesting that any compromise of mesothelial health may actually reduce its ability to induce ESC coupling which is required for invasion. These studies provide the first direct evidence that the pathological changes underlying the invasive behavior that allows lesions to form in endometriosis arise initially in the endometrium, indicating that it should be the primary target for therapy or diagnosis of the disease.

## Figures and Tables

**TABLE 1:** Demographics of contributing patients.

Age (y)	Race	BMI (kg/m^2^)	Type	PMC	ESC
31	Hispanic	39.6	Control	X	X
30	White	38.2	Control	X	X
36	White	33.1	Control	X	—
28	White	37.3	Control	X	—
25	Hispanic	27.7	Control	X	X
33	White	38	Control	X	—
29	Hispanic	24.5	Control	X	—
40	Hispanic	29.2	Control	—	X
30	Hispanic	25.8	Stage 1 endometriosis	X	X
28	White	21.7	Stage 2 endometriosis	X	—
25	Hispanic/Pacific Islander	24.2	Stage 2 endometriosis	X	—
34	Hispanic	18.9	Stage 3 endometriosis	X	—
32	White	23	Stage 3 endometriosis	X	X
34	White	21	Stage 3 endometriosis	X	X
25	White	40.2	Stage 1 endometriosis	X	—
27	Hispanic	32.8	Stage 1 endometriosis	—	X

*Note:* BMI = body mass index; ESC = endometrial stromal cell; PMC = primary mesothelial cell.

## F&S SCIENCE CLINICAL QUICK TAKE

### What clinical problem is addressed by these studies?

- Endometriosis affects 10% of all women, yet the origin of disease remains obscure, including whether it arises from uterine or peritoneal environment. This work establishes the cause firmly with the endometrium.

### What are the key findings?

- Using both primary stromal and mesothelial cells from patients with and without endometriosis, we demonstrated that stromal cell origin was the main determinant of endometrial cell invasion across mesothelial cells, the first step in endometriosis lesion formation.

### How do these findings apply to human fertility or the reproductive process?

- Endometriosis contributes to 50% of infertility worldwide, and the current studies now demonstrate that the origins of the disease are, indeed, in the endometrium, consistent with the loss of fertility.
